# Biopesticides and Human Health Risks: A Critical Review

**DOI:** 10.3390/toxics14030246

**Published:** 2026-03-11

**Authors:** Sandra Petrović, Andreja Leskovac

**Affiliations:** Vinča Institute of Nuclear Sciences—National Institute of the Republic of Serbia, University of Belgrade, M. Petrovića Alasa 12-14, 11351 Belgrade, Serbia; sandra@vin.bg.ac.rs

**Keywords:** botanical biopesticides, human health, risk perception, regulatory framework

## Abstract

The transition toward more sustainable crop protection under the European Green Deal has accelerated the adoption of biopesticides, which are widely considered safer alternatives to synthetic pesticides. Botanical biopesticides derived from plant extracts, essential oils, and secondary metabolites are increasingly used in both conventional and organic agriculture. However, their growing use raises important questions regarding human health risks. Botanical biopesticides are complex mixtures of bioactive compounds whose composition and toxicological profiles can vary substantially depending on plant chemotype, extraction method, and manufacturing processes. This review critically examined the toxicological properties of botanical biopesticides and evaluated their regulatory assessment within the European Union (EU) framework. Particular attention is paid to scientific uncertainties, gaps in toxicological data, challenges in hazard characterization of complex mixtures, and limitations of current human exposure assessments. The review also considered how regulatory practices, user behavior, and risk perception may influence real-world exposure and potential health outcomes. By integrating experimental toxicology studies, EU risk assessment documents, and evidence on agricultural use patterns, this review assessed whether reduced intrinsic toxicity of botanical biopesticides translates into lower human health risk under current regulatory frameworks and agricultural practices. The findings underscore the need for strengthened toxicological evidence, harmonized regulatory approaches, and improved risk communication to ensure that the use of botanical biopesticides remains aligned with good agricultural practice and human health protection.

## 1. Introduction

The transition toward more sustainable crop protection is a central part of the European Green Deal and the Farm-to-Fork Strategy, which call for a 50% reduction in chemical pesticide use by 2030 and for at least 25% of the European Union (EU) agricultural land to be under organic farming [1]. Within this regulatory framework, biopesticides are widely promoted as safer and more environmentally friendly alternatives to conventional synthetic pesticides. Synthetic pesticides exhibit rapid, broad-spectrum pest control and consistent performance, making them cost-effective for large-scale agriculture [2]. However, their environmental persistence and the potential adverse effects on non-target organisms pose significant environmental and health concerns [3]. These limitations have increased demands for biopesticides whose natural origin, target specificity, biodegradability, and perceived safety have led to greater interest among policymakers, farmers, and consumers [4]. Biopesticides are pest control products derived from natural sources, including microorganisms, botanical materials, and plant secondary metabolites [5]. They are generally classified into three main categories: (i) microbial biopesticides, which include bacteria, fungi, viruses, or protozoa, (ii) biochemical biopesticides, which encompass plant-derived substances, pheromones, and other naturally occurring compounds, and (iii) plant-incorporated protectants, in which plants are engineered to produce their own protective agents [6]. Although they still represent a relatively small portion of the global pesticide market, biopesticides are experiencing rapid expansion. In 2024, the global biopesticide market was valued at approximately USD 7.8 billion and is projected to reach nearly USD 32 billion by 2034 [7].

Botanical biopesticides, which fall under the category of biochemical biopesticides, are derived from plant extracts, essential oils, and other plant secondary metabolites [6]. They are expanding relatively fast, reflecting rising market demand and heightened interest in their potential human health and environmental impacts. The increased use of botanicals is closely linked to the perception that most natural plant-derived products pose minimal risk [8]. However, this assumption warrants critical evaluation, as botanicals are complex mixtures of bioactive compounds with diverse, and in many cases, insufficiently characterized toxicological profiles [9]. Their composition can vary substantially depending on plant chemotype, extraction method, environmental conditions, and manufacturing processes [10,11], complicating hazard identification, toxicological assessment, and regulatory decision-making.

The European Union has established a comprehensive and highly structured regulatory framework governing the approval, classification, and monitoring of pesticides. This system integrates pre-market risk assessment, post-authorization monitoring, and periodic re-evaluation to ensure that plant protection products (PPPs) meet rigorous standards for human health, environmental safety, and food-chain protection. The approval of botanical active substances is governed by the same regulatory framework applied to synthetic PPPs under Regulation (EC) No 1107/2009 [12]. As part of this process, the European Food Safety Authority (EFSA) conducts scientific risk assessments that underpin regulatory decisions, evaluating the potential impacts of biopesticides on human health, non-target organisms, and the environment. This multi-layered approach ensures that pesticides, including biopesticides, cannot be approved without robust evidence that human exposure remains below health-protective thresholds. An active substance may be approved when it is considered safe for human health and unlikely to cause unacceptable environmental impacts. However, in some cases, plant-derived active substances have been approved although regulatory evaluations highlight data gaps and scientific uncertainties that limit the complete characterization of their safety. Limitations in testing methodologies, gaps in available data, and obligations to communicate the adverse effects of pesticides (i.e., post-marketing surveillance) imply that some harmful effects may only become evident after prolonged use [13]. During the renewal process, active substances remain available on the market, even though new risk assessments are carried out. In such cases, parts of the risk assessment rely on incomplete data sets, reflecting areas where further evidence is required to establish safety under all relevant exposure scenarios. However, historically, this has sometimes led to the continued use of substances later proposed for ban, such as natural products like nicotine and rotenone [14].

Beyond regulatory and toxicological uncertainties, human factors introduce an additional aspect to the safety assessment of biopesticides. Biopesticides are underlined in the scientific literature as environmentally preferable and sustainable alternatives to conventional pesticides [2,9,15,16], an emphasis that substantially shapes user perceptions. In addition, reports from the European Parliament and the Institute for European Environmental Policy frame biopesticides as a core component of a more sustainable agricultural future, aligning use of these technologies with broader Common Agricultural Policy (CAP) and Green Deal objectives [17,18]. While such narratives emphasize the environmental benefits of plant-derived products, they may also unintentionally reinforce the perception among farmers and consumers that plant-derived products are inherently safe. This perception can, in turn, result in inadequate risk-management practices, including insufficient use of personal protective equipment (PPE), relaxed adherence to application guidelines, or increased treatment frequency beyond recommended limits. Such behaviors can elevate occupational exposure, particularly among small-scale or insufficiently trained operators, and potentially offset the presumed safety advantages of botanical formulations. Moreover, limited systematic monitoring of operator, bystander, and consumer exposure, as outlined in existing regulatory guidance [19], combined with the scarcity of epidemiological evidence, contributes to persistent uncertainty regarding the real-world human health risks associated with both synthetic and botanical pesticides.

This review evaluated the human health risks associated with botanical biopesticides and examined their regulatory status in the EU. Scientific uncertainties, toxicological data gaps, regulatory challenges, and practical issues related to user behavior and risk perception are briefly discussed. By integrating toxicological evidence, EU risk assessment documents, and studies on agricultural and consumer practices, this review examined the extent to which botanical biopesticides can be considered safer for human health and whether perceptions linked to their natural origin may influence risk assessment, risk communication, and user practices.

References included in this review, published up to December 2025, were identified through a structured search of scientific databases, including Web of Science, Scopus, PubMed, ScienceDirect, and Google Scholar. Peer-reviewed articles published in English addressing biopesticide chemistry, efficacy, toxicity, human health risk, and regulatory frameworks were prioritized. Additionally, pertinent grey literature, such as government agency reports and reputable market analyses, was included to provide a comprehensive perspective.

## 2. Regulatory Framework for Biopesticides in the European Union

Given the expanding use of botanical biopesticides, understanding the European regulatory framework is essential for evaluating how these products are authorized, monitored, and classified as low-risk alternatives to conventional pesticides.

As noted, within the EU, biopesticides are regulated under the same legislative framework as synthetic PPPs. Their approval follows the procedures outlined in Regulation (EC) No 1107/2009, with Regulation (EC) No 396/2005 setting legal limits on pesticide residues in food and feed, which requires a comprehensive assessment of efficacy, toxicology, residues, environmental fate, and ecotoxicology before an active substance can be approved for use [12,20]. In this process, the EFSA plays a central role by conducting scientific risk assessments that inform regulatory decisions. In addition, biopesticides must meet the data requirements set out in Regulation (EU) No 283/2013 (active substances) [21] and Regulation (EU) No 284/2013 (formulated products) [22]. EFSA is pivotal in setting and reviewing maximum residue levels (MRLs) for pesticides in food and feed marketed in the EU, based on detailed toxicological and exposure assessments [23]. Its evaluation of pesticides is among the most extensive globally, covering acute and chronic toxicity, carcinogenicity, genotoxicity, reproductive and developmental toxicity, endocrine disruption, neurotoxicity, immunotoxicity, dietary exposure, and cumulative effects [24,25]. Dietary exposure through food and drinking water is assessed against toxicological reference values, such as the acceptable daily intake (ADI) and the acute reference dose (ARfD) [24].

As agricultural practices increasingly involve multiple pesticide applications, EFSA has expanded its focus to the risks posed by combined exposures [26]. Over the past decade, EFSA has developed cumulative risk assessment (CRA) methodologies that evaluate the joint effects of pesticides sharing a common toxicological mode of action. Substances are organized into Cumulative Assessment Groups (CAGs), and combined exposures from food and water are compared to health-based thresholds using approaches such as the Margin of Exposure (MOE) [26,27,28]. The EFSA and the European Commission have introduced a phased plan to implement CRA for pesticides into EFSA’s and Member States’ regulatory activities starting in 2022 [29]. According to this roadmap, all toxicological endpoints relevant for CRA are expected to be fully identified by 2030, and CAGs for each target organ or physiological system will be established. To date, EFSA has completed several retrospective CRAs, including assessments of chronic thyroid effects [30], acute and chronic impacts on the nervous system [31,32], and acute craniofacial developmental alterations [33]. Ongoing work focuses on cumulative risks affecting the liver, kidneys, and the reproductive system. These retrospective evaluations need periodic updating to reflect changes in exposure patterns and possible revisions to the underlying CAGs. The CRA implementation plan aligns with the broader Risk Assessment of Combined Exposure to Multiple Chemicals (RACEMiC) initiative, which seeks to harmonize combined-exposure assessment approaches across all EFSA sectors [34]. Mixture risk assessment (MRA) remains scientifically challenging, particularly where data on additive, synergistic, or antagonistic interactions are limited. Regulation (EC) No 396/2005 requires that cumulative and synergistic effects be considered in MRLs settings once validated methods are available, underscoring the need for continued methodological development [20]. Advances in residue detection, degradation modelling, and exposure assessment tools are therefore essential. Recent updates, including refinements to the Monte Carlo Risk Assessment (MCRA) platform, aim to increase transparency and improve the robustness of cumulative and mixture risk evaluations [35].

MRLs apply to all PPPs placed on the EU market, including biopesticides. Still, many botanical and microbial substances are exempt because they leave no detectable or relevant residues under regular use. Regulation (EC) No 396/2005 specifies that MRLs must be established unless a substance qualifies for inclusion in Annex IV, reserved for active substances considered to pose no consumer health risk, such as some microorganisms and plant extracts with negligible toxicity. For botanical biopesticides that produce identifiable residues, however, standard residue definitions, metabolism data, and supervised field trials are still required. [20,36]. Botanical biopesticides are particularly relevant to organic agriculture, where the use of synthetic pesticides is highly restricted. Under Regulation (EU) 2018/848 on organic production, only PPPs whose active substances are approved under Regulation 1107/2009 and listed as compatible with organic farming may be used [37]. Therefore, organic farming relies on biopesticides that meet both the regulatory safety criteria set by EFSA and the principles of organic production, including environmental protection and minimal synthetic inputs. Moreover, organic agriculture often functions as an early adopter of such products, stimulating market demand and supporting the EU’s broader pesticide-reduction goals under the Farm to Fork Strategy. Collectively, this framework ensures that biopesticides undergo rigorous, science-based evaluation, enabling their implementation as safer alternatives in sustainable agriculture.

Although botanical biopesticides are widely used in organic farming, they are not limited to organic agriculture. Directive 2009/128/EC on the sustainable use of pesticides promotes the incorporation of low-risk substances within Integrated Pest Management (IPM), encouraging Member States to reduce reliance on chemical pesticides [38]. Thus, under EU law, biopesticides are also used in conventional farming [2,39]. Conventional producers use biopesticides to reduce chemical residues, manage resistance to synthetic pesticides, and meet stricter environmental or market requirements [40]. For example, azadirachtin, pyrethrin, orange oil, and clove oil are widely used in conventional fruit, vegetable, and crop production [41]. Therefore, biopesticides function as cross-sector products, used across all production systems.

Global demand for biopesticides has increased steadily in parallel with consumer interest in organic and low-residue foods, creating intense market pressure for alternatives to synthetic pesticides. Studies predict that biopesticides will reach the market size of synthetic pesticides by the late 2040s or early 2050s, indicating a significant growth trajectory [42]. Worldwide, more than 1400 biopesticide products have been registered [43]. However, the number of approved products in the EU remains comparatively low due to its more demanding regulatory framework [44]. At the same time, demand for organic agriculture has expanded rapidly in line with policy objectives under the Farm to Fork Strategy, aiming to have 25% of agricultural land organic by 2030 [1]. Between 2012 and 2020, the share of agricultural land under organic farming increased by more than 50%, reaching approximately 9.1%, while organic retail sales nearly doubled between 2015 and 2020 [45]. By market value, the United States (EUR 58.6 billion), Germany (EUR 15.3 billion), and China (EUR 12.4 billion) accounted for the largest organic markets in 2022. Globally, organic product sales reached EUR 134.8 billion in 2022, an almost ninefold increase compared with EUR 15.1 billion in 2000. Europe accounted for EUR 53.1 billion of these sales, making it the second-largest organic market worldwide, following the United States [46].

**Figure 1:**
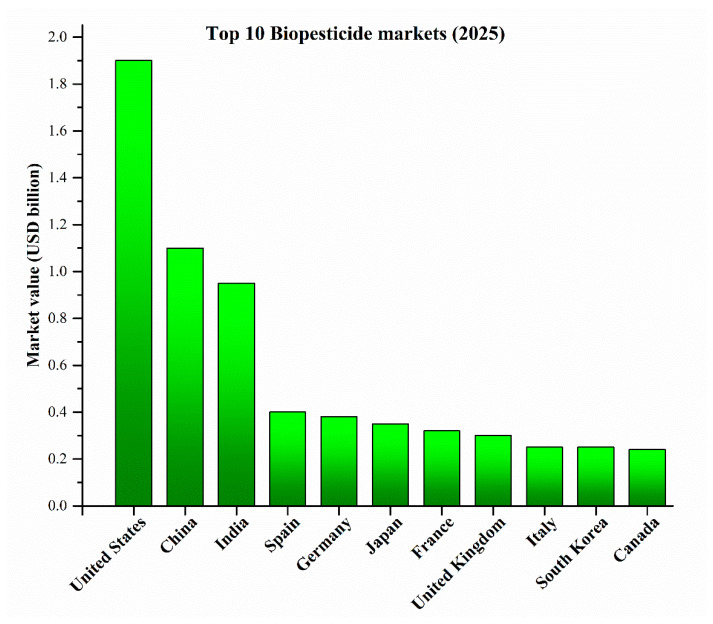
Estimated biopesticide market values (USD billion) by country in 2025. Market values are derived from industry estimates; equal values (Italy and South Korea) result in shared ranking positions. Data adapted from IndustryResearch.biz [47].

As per the data of IndustryResearch.biz. [47], Europe accounts for a substantial share of the global biopesticide market, representing approximately 20.8% of worldwide sales in 2024. Germany holds the largest share of the European market (nearly 29%), followed by France (23%), Italy (16%), Spain (15%), and the Netherlands (10%). Increased adoption is also evident in Southern Europe. Globally, as shown in Figure 1, based on regional market data, the largest national biopesticide markets in 2025 are estimated to be the United States, China, India, Spain, and Germany, reflecting strong adoption of biopesticides across diverse agricultural sectors [47].

Regulatory approaches to biopesticides differ substantially between the United States and the European Union, leading to contrasting market dynamics and approval timelines. In the U.S., the simplified registration submission process and reduced registration fees by the Environmental Protection Agency (EPA) result in significantly faster authorizations and a larger biopesticide market [48]. By contrast, since the EU assesses biopesticides under the same regulatory framework as conventional pesticides, it requires considerably longer approval times and higher costs for applicants [2]. As a result, the U.S. marketplace hosts several hundred registered biopesticide products, while the EU authorizes only a portion of them [49]. For instance, several botanical biopesticides, such as tea tree oil, eucalyptus oil, jojoba oil, cinnamon oil, and capsaicin (hot pepper extract), widely used in the U.S, are not approved as PPPs in the EU [50]. Examples of EU-approved botanical biopesticides are presented in Table 1.

The slow pace of the EU’s regulatory approval process continues to widen the gap between market needs and product availability. Numerous researchers point out that the current EU regulatory framework was initially developed for synthetic pesticides rather than biopesticides, whose properties, modes of action, and environmental fate differ significantly [51,52].

**Table 1:** Examples of EU-approved botanical PPPs.

Botanical PPP/Origin	Major Constituents	Main Activity	Formulation Type/Product Example	Reference
Azadirachtin (Margosa extract) (*Azadirachta indica*)	limonoids: Azadirachtin A (the main component), B, and H, Azadiradione, 3-desacetyl-salannin, 6-desacetyl-nimbin, 11-epiazadirachtin D, Nimbin, Ohchinolide B, Salannin, 14,15-epoxy-azadiradione	Insecticide Acaricide	EC; NeemAzal-T/S, Oikos	[53]
Pyrethrins (pyrethrum) (*Tanacetum cinerariifolium*)	esters of chrysanthemic acid: Pyrethrin 1 and 2, Cinerin 1 and 2, Jasmolin 1 and 2	Insecticide	EC; Pirecris, Pyrethrum 5EC, Evergreen Growers Spray 7439	[54]
Clove oil (*Syzygium aromaticum*)	eugenol, methyl eugenol, β-caryophyllene (or caryophyllene), α-caryophyllene (or humulene), caryophyllene oxide, eugenol acetate, meta eugenol, δ-cadinene, and calamenene	Insecticide Herbicide Fungicide Nematicide Rodenticide Bactericide	EC; Bioxeda	[55]
Orange oil (peel oil) (*Citrus aurantium var.* *Dulcis*)	complex mixture of terpenes; D-limonene (the main component)	Insecticide Herbicide Repellent	ME; Prev-Am	[56]
Rape seed oil (*Brassica napus*)	mixture of triglycerides of fatty acids	Insecticide, Acaricide	EC; NEU 1160 I	[57]
Spearmint oil (*Mentha spicata, Mentha* *Cardiaca*)	complex mixture; (R)-carvone (the main constituent)	Plant growth regulator Insecticide Fungicide,	HN; BIOX-M	[58]
Citronella oil * (*Cymbopogon nardus*)	citronellal, geraniol, citronellol, geranyl acetate	Herbicide	EW; Barrier H	[59]
Eugenol (*Syzygium aromaticum*)	phenol eugenol	Insecticide Herbicide Fungicide	CS; Mevalone 3AEY, (3.2% eugenol, 6.4% geraniol, and 6.4% thymol)	[60]
Thymol (*Thymus vulgaris*)	monoterpene phenol thymol	Fungicide Bactericide		[61]
Geraniol (*Cymbopogon martinii, Monarda fistulosa*)	Monoterpene geraniol	Fungicide		[62]
Garlic extract (*Allium sativum*)	diallyl sulfide, diallyl disulfide, diallyl trisulfide, and diallyl tetrasulfide (marker compounds)	Repellent Insecticide Nematicide	NEMguard SC/ECOguard SC, NEMguard Granules (GR)/ECOguard Granules (GR)	[63]
*Quassia amara* wood extract *(Quassia amara* L.)	Mixture; quassin (the main component), neoquassin, isoquassin, parain, quassimarin, quassino	Insecticide Repellent	SC; pure *Quassia amara* L. wood for herbal infusion preparation	[64]

* Citronella oil was approved in the EU and temporarily included in Annex IV of Regulation (EC) No 396/2005; however, the renewal application was withdrawn, and its approval expired. EC—emulsifiable concentrates, CS—capsule suspension, ME—micro-emulsion, HN—Hot fogging concentrate, EW—oil in water emulsion, SC—suspension concentrate, GR—granule formulation.

As a result, various expert groups call for a reformed system that distinguishes biopesticides from conventional pesticides, simplifies the registration and authorization process, and unifies biopesticide-specific legislation to accelerate approvals and support the EU’s green transition objectives [9,52,65]. In this context, the European Parliament adopted the “Report on Ensuring the Faster Registration and Uptake of Biological Control Agents” (A10-0234/2025), within procedure 2025/2086 (INI). The report highlights the urgent need to modernize the EU pesticide approval system to facilitate the authorization and uptake of biocontrol agents, while maintaining high safety standards for health and the environment. The European Parliament calls on the Commission to define biological control solutions in EU law and to establish a framework for the accelerated approval of these substances and products [66]. This approach balances innovation with precaution, supporting the transition toward safer crop protection without compromising human and environmental health.

Although improving regulatory assessment is a legitimate objective, accelerating authorization timelines without ensuring sufficiently robust toxicological evaluation may undermine the fundamental rationale for adopting biopesticides: reducing health and environmental risks. Plant-derived substances are not inherently nontoxic, and EFSA evaluations have, in several cases, identified concerns related to skin and respiratory sensitization, potential endocrine or reproductive effects, and uncertainties regarding impurities, metabolites, and degradation products. Thus, although regulatory reform is necessary to accommodate technological innovation and market demands, maintaining scientifically rigorous, risk-based health assessments must remain the primary criterion to ensure that efforts to streamline approvals do not compromise scientific rigor or public safety.

## 3. Chemical Variability and Toxicity of Botanical Biopesticides

Sustainable agriculture increasingly relies on botanical biopesticides to address environmental, health, and resistance concerns associated with conventional pesticides [67]. However, their use must be interpreted in light of inherent chemical variability, which can influence toxicological profiles and exposure patterns. This chemical complexity underscores the need for systematic evaluation of toxicity and potential human health risks. Accordingly, key toxicological aspects of selected botanical biopesticides, with emphasis on issues most relevant to current regulatory and safety assessments, are briefly discussed.

### 3.1. Chemical Variability of Botanical Biopesticides

Numerous plant families, such as Myrtaceae, Lauraceae, Rutaceae, Lamiaceae, Asteraceae, Apiaceae, Cupressaceae, Poaceae, Zingiberaceae, Piperaceae, Liliaceae, Apocynaceae, Meliaceae, Solanaceae, Caesalpinaceae, and Sapotaceae, are reported to produce bioactive constituents with pesticidal activity [68]. Botanical biopesticides act through secondary metabolites, including alkaloids, rotenoids, phenolics, pyrethrins, various oils, and saponins, which confer antibacterial, antifungal, herbicidal, insecticidal, and nematicidal properties [69]. Essential oils (EOs) and plant extracts are the most widely used forms of botanical biopesticides [9]. Plant extracts are generally prepared from dried plant material by solid–liquid extraction with aqueous or organic solvents, yielding mixtures rich in alkaloids, saponins, and sterols [9]. EOs are primarily obtained by steam distillation, although methods such as fermentation, solvent extraction, and enfleurage are also used [70]. The compositional variability of botanical biopesticides is strongly influenced by extraction methodology. Steam distillation and hydrodistillation preferentially isolate volatile compounds such as monoterpenes and other terpenoids. However, high temperatures can cause thermal degradation or loss of heat-sensitive constituents, resulting in extracts with distinct profiles and potentially different exposure risks than the original plant material [71]. In contrast, solvent extraction tends to retain a broader spectrum of volatile and non-volatile phytochemicals, including phenolics, alkaloids, and less volatile secondary metabolites, which may alter systemic toxicity profiles and biological activity due to the presence of diverse bioactives and organic solvent residues [72]. Supercritical CO_2_ extraction (scCO_2_) can selectively concentrate nonpolar fractions at lower temperatures, often yielding higher purity and enhanced bioactivity compared to traditional methods. Nevertheless, differences in polarity and co-solvent use can also yield distinct chemical profiles compared to those of steam-distilled or solvent extracts [73]. Studies comparing extraction methods in aromatic plants have demonstrated significant variation in the yields, compositions, and bioactivities of isolates, indicating that the extraction technique can influence both the chemical composition and the biological effects of botanical extracts [74,75]. These methodological differences may substantially modify hazard classification, sensitization potential, and other toxicological properties even when derived from the same botanical source.

EOs are typically obtained from flowers, leaves, roots, or seeds and consist of complex mixtures of volatile secondary metabolites, such as monoterpenes (e.g., D-limonene, α-pinene, myrcene, eucalyptol), terpenes (e.g., thymol, carvacrol), terpene alcohols (e.g., geraniol, linalool), terpene aldehydes (e.g., citral, citronellal), ketone-containing terpenes (e.g., carvone), phenolic phenylpropanoids (e.g., eugenol), terpene oxides, sesquiterpenes and nitrogen- or sulfur-containing compounds, which can contribute to bioactivity and pesticidal properties [76,77].

Through some of these metabolite classes, EOs and crude plant extracts function as broad-spectrum insecticides and may act as repellents, attractants, antifeedants, or respiratory inhibitors [9]. They can disrupt host-plant recognition, deter oviposition, and exhibit ovicidal or larvicidal activity [44]. Within this broader category, limonoids represent a structurally distinct group of highly oxygenated triterpenoids primarily found in plant species of the Meliaceae (e.g., *Azadirachta*, *Melia*) and Rutaceae (e.g., *Citrus*) families [9]. Azadirachtin, the main limonoid from *Azadirachta indica* (neem), is one of the most-studied botanical insecticides, widely used in biopesticide formulations due to its potency at very low concentrations. Similarly, pyrethrins, esters derived from the flowers of *Tanacetum cinerariifolium*, act as fast-acting neurotoxins that target insect sodium channels [78].

Empirical evidence further demonstrates that crude plant extracts from genera such as *Allium*, *Zingiber*, *Curcuma*, *Citrus*, *Azadirachta*, *Phyllanthus*, *Calotropis*, and *Ocimum*, in combination with microbial antagonists from *Trichoderma* and *Paecilomyces*, can effectively suppress major insect pests, including whiteflies, thrips, and *Helicoverpa* spp. They also reduce the incidence of fungal diseases caused by *Uromyces*, *Phaeoisariopsis*, and *Colletotrichum* species in crops such as green beans and tomatoes [70]. The insecticidal action of botanical biopesticides is multifaceted, depending on their chemical composition, the insect pest species, and its developmental stage [79]. Specifically, the insecticidal effects of EOs or plant extracts are primarily mediated through interactions with the insect nervous system. Documented mechanisms include modulation of γ-aminobutyric acid (GABA)-gated chloride and sodium channels, inhibition of acetylcholinesterase (AChE), interference with nicotinic acetylcholine receptors (nAChR), and interference with octopamine and tyramine receptors [79].

Essential oils rich in monoterpenes and phenolic derivatives, such as α-pinene, β-pinene, farnesene, eugenol, eucalyptol, juglone, camphor, limonene, pulegone, menthol, menthone, citral, carvacrol, carvone (R/S), trans-caryophyllene, thymol, geraniol, and citronellol have been reported to exhibit herbicidal or allelopathic activity, affecting seed germination, root growth, and plant development in target species [80]. Beyond individual compounds, numerous EOs exhibit herbicidal effects that are likely mediated by synergistic interactions among their diverse constituents. Examples include EOs from *Artemisia fragrans*, *Artemisia scoparia*, *Cymbopogon citratus*, *Citrus aurantiifolia*, *Cinnamomum zeylanicum*, *Cymbopogon winterianus*, *Heterothalamus psiadioides*, *Hyptis suaveolens*, *Mentha longifolia*, *Mentha x piperita*, *Monarda didyma*, *Nepeta nuda*, *Origanum vulgare*, *Plectranthus amboinicus*, *Pogostemon benghalensis*, *Salvia leucophylla*, *Syzygium aromaticum*, and *Vitex negundo* [81]. Herbicidal activity of EOs and their bioactive constituents functions through several physiological and biochemical mechanisms. As discussed by Werrie et al. [80], Gruľová et al. [81], and Motmainna et al. [82], they can (i) compromise the integrity of the cuticle, leading to desiccation and tissue damage, and interfere with core metabolic processes by inhibiting photosynthesis and mitochondrial respiration; (ii) modulate enzymatic pathways and phytohormone signaling, disrupt cellular water balance, and alter membrane structure and function, thereby impairing nutrient transport and cellular homeostasis; (iii) interfere with cytoskeletal dynamics, disrupting microtubules and potentially causing genotoxic effects, or (iv) stimulate the production of reactive oxygen and nitrogen species, resulting in oxidative stress. Collectively, these mechanisms contribute to growth inhibition, tissue necrosis, and plant death, underscoring the complex herbicidal potential of botanical biopesticides [83].

The diversity of botanical sources and their secondary metabolites supports their broad use as biopesticides, but this same diversity also leads to considerable mechanistic complexity. Understanding the mechanisms underlying their pesticidal effects is essential for evaluating their efficacy, optimizing formulations, and predicting their potential risks. Although these mechanisms are well characterized, predicting biological outcomes remains challenging because botanical products exhibit substantial intrinsic variability in their chemical composition [84]. Consequently, the biological potency, mode of action, and toxicological properties of botanical biopesticides are far less consistent than those of synthetic pesticides. Unlike synthetic pesticides, typically composed of single active molecules, botanical formulations are complex mixtures of secondary metabolites whose concentrations fluctuate with plant chemotype, growth stage, extraction method, storage conditions, and environmental factors such as soil type, cultivation conditions, harvest time, geographic and ecological locations, and climate [14,85]. For instance, even within a single plant species, secondary metabolite production and essential oil composition can vary markedly depending on the genotype or chemotype [86,87,88]. Moreover, EOs may contain dozens of bioactive constituents that exhibit synergistic or antagonistic interactions, making it challenging to attribute biological effects to a specific compound or to predict interactions reliably [89,90].

Furthermore, soil type, light irradiation (duration, intensity, quality), temperature, climate, altitude, and other environmental parameters influence secondary metabolite biosynthesis [10]. Such variability may result in biopesticide products derived from the same plant species displaying substantially different toxicological properties, activity spectra, environmental persistence, and effects on non-target organisms, thereby contributing to inconsistent efficacy, batch-to-batch variations, and limited predictability of their toxicological profiles [14,91,92]. These challenges complicate not only the standardization of commercial products but also the reproducibility of toxicological testing and human exposure assessments.

### 3.2. Toxicity of Botanical Biopesticides

Toxicity profiles of botanical biopesticides vary significantly depending on the plant source, extraction method, formulation, dose, and exposure route. Although acute toxicity of botanicals is often reported as low, data on chronic exposure, endocrine disruption, neurotoxicity, developmental toxicity, and genotoxicity remain limited or inconsistent [14,93]. Botanical biopesticides may contain bioactive constituents associated with skin sensitization, respiratory or contact allergies, reproductive and developmental toxicity, and, in some cases, carcinogenic potential, particularly when specific components undergo metabolic activation or exert endocrine-modulating effects [83]. EOs most frequently implicated in allergic reactions include those derived from turpentine-producing pines, tea tree, ylang-ylang, lavender, peppermint, rose, jasmine absolute, and sandalwood [94]. Furthermore, some EO constituents, including anethole, citral, camphor, thujone, and pulegone, have been shown to affect reproductive hormones and induce fetotoxic or embryotoxic effects [95]. Considering carcinogenic potential, most EOs are not inherently carcinogenic; however, certain constituents can act as secondary carcinogens following metabolic activation. For example, *Salvia sclarea* and *Melaleuca quinquenervia* oils may stimulate estrogen production, potentially promoting estrogen-dependent cancers [83]. Photosensitizing compounds such as psoralen, flavins, cyanine, and porphyrins can increase the risk of skin cancer, while pulegone, safrole, methyleugenol, D-limonene, and estragole can form carcinogenic metabolites in rodents [83,96].

Experimental studies in animals indicate that several compounds, including rotenone, pyrethrins, nicotine, and azadirachtin, can induce hepatotoxicity, renal toxicity, neurotoxicity, reproductive toxicity, oxidative stress, and genotoxic or mutagenic effects, with outcomes depending on chemical composition, dose, exposure duration, and the target organism [93]. Historically, early botanical insecticides, such as nicotine and rotenone, were withdrawn from the European market due to mammalian toxicity [14]. Nicotine, a tobacco alkaloid, is highly insecticidal but non-selective, with an oral lethal dose of 50–60 mg in humans, while rotenone, a flavonoid from Fabaceae species, disrupts mitochondrial respiration and has been associated with Parkinson’s disease risk [14,97,98].

For several botanical PPPs approved in the EU, the availability of toxicological reference values, such as ADI, ARfD (dietary exposure), or acceptable operator exposure level (AOEL) (non-dietary exposure), remains inconsistent across substances. In some cases, these reference values have not been established due to insufficient toxicological data or because exposure was considered negligible under proposed conditions of use. Table 2 summarizes the impact on human health and toxicological reference values for some botanical biopesticides, which are selected based on the following criteria: (i) current regulatory approval within the EU framework, (ii) representation of chemically distinct classes of plant-derived bioactive compounds, and (iii) documented agricultural use across different application sectors. For several botanical biopesticides, the assessment is largely driven by data on main constituents and representative uses, and long-term datasets may be less extensive than those required for conventional synthetic pesticides.

Pyrethrins are widely used botanical insecticides, and although generally considered safer than many synthetic pesticides, they still present notable risks for human and mammalian health. Their primary mode of action is to interfere with neuronal sodium channels, leading to overstimulation of the nervous system. Pyrethrins have demonstrated genotoxic effects in murine models and cytotoxicity in human HepG2 and SH-SY5Y cells [103]. They can also provoke dermatitis and, in cases of ocular exposure, corneal erosions [104]. Overall, pyrethrins exhibit low acute toxicity, though large ingestions may cause convulsions, and rare fatalities from acute asthma have been reported [104]. EFSA’s evaluation of pyrethrins found that they are unlikely to be genotoxic. Inhalation toxicity is supported by a 90-day rat study. Concerns were identified regarding neurotoxicity, developmental toxicity, and potential endocrine-mediated effects. Data on residues were insufficient to establish a reliable consumer risk assessment, and environmental exposure remains incompletely characterized. Generally, several key data gaps regarding residues, metabolites, and environmental exposure persist [54].

Another widely used botanical insecticide, azadirachtin, is generally characterized by low acute toxicity to mammals. However, in vitro studies have demonstrated that azadirachtin can induce cytotoxic and genotoxic effects in mammalian cells at high doses [93]. Available evidence suggests that prolonged exposure to neem-derived products may pose risks to human health, particularly when consumed orally or used improperly. Both intentional and accidental ingestion have been associated with clinical symptoms ranging from mild to severe vomiting, drowsiness, seizures, metabolic acidosis, convulsions, and encephalopathy, with some cases documented as potentially life-threatening in both children and adults [105,106,107,108,109]. Although azadirachtin exhibits low acute toxicity and minimal risk to humans under expected use conditions, EFSA highlighted significant uncertainties arising from the intrinsic chemical complexity of Margosa (neem) extracts [53]. The EFSA assessed azadirachtin for its use as an insecticide on potatoes and as an acaricide on greenhouse ornamentals. Azadirachtin A was confirmed as the lead active component with sufficient efficacy, and non-dietary exposure for workers, bystanders, and residents in permanent greenhouses was considered low. However, consumer risk assessment remains uncertain due to incomplete data on residues, breakdown products, and the relative toxicity of other extract components. Environmental fate and behavior data were partially sufficient, but gaps remain regarding groundwater exposure, residue definitions, and aquatic risk assessment for the proposed ornamental use. The risk to non-target organisms was generally low for greenhouse applications, although further clarification is needed for aquatic organisms, soil organisms, and non-target arthropods under broader use scenarios [53].

EOs, primarily composed of monoterpenes that target insect octopaminergic systems, are generally considered safe for mammals. Nevertheless, excessive doses may induce neurotoxic, cytotoxic, and genotoxic effects, as well as modulate estrogen production [14]. Some of these EO phenolic compounds have demonstrated cytotoxic and genotoxic effects in mammalian cells in vitro [83]. For example, phenolic compounds such as eugenol, isoeugenol, methyleugenol, and safrole can trigger apoptosis, necrosis, and DNA damage in rodent hepatocytes. Estragole has been reported to induce unscheduled DNA synthesis in V79 hamster fibroblasts [96]. Additionally, carvacrol, thymol, and eugenol, which are generally considered safe and widely used in food and non-food applications, have emerged as potential toxicants as reported in several in vitro and in vivo studies [110]. In animal studies, acute and prolonged exposure to carvacrol and thymol has caused adverse effects in mice, rats, and rabbits [111], and eugenol has induced pulmonary and renal damage in exposed frogs [112,113]. In humans, exposure to these compounds may lead to skin irritation, inflammation, dermatitis, ulcer formation, allergic reactions, liver and kidney damage, and other health issues [110,114,115,116,117].

In recent years, EFSA has released several reports on thymol, covering its use as a food flavoring, an animal feed additive, and a pesticide. Thymol was approved as a PPP with several confirmatory data requirements, including long-term and reproductive toxicity, comparisons of natural background exposure with exposure from pesticidal uses, and groundwater assessment. Additionally, consumer exposure from pesticide use could not be reliably compared with natural background exposure. The groundwater exposure was considered unlikely to exceed regulatory thresholds, but uncertainties remained for ecotoxicological risk assessment, particularly for birds, mammals, and aquatic organisms. Therefore, key uncertainties in toxicology, consumer dietary exposure, and ecotoxicology still preclude a complete and reliable risk characterization [61].

EFSA’s peer review of eugenol identified several major concerns and data gaps [60]. The genotoxic and carcinogenic impurity methyleugenol remains a key concern, with no health-based reference values established, preventing a reliable evaluation of consumer dietary exposure. Residue data are limited by insufficient information on storage stability and incomplete analytical methods for methyleugenol in treated crops. Groundwater contamination risk appears low under typical use, but ecological risks to birds and aquatic organisms remain uncertain due to insufficient data to compare natural and pesticidal exposures. Overall, the assessment highlights the need for additional toxicological and residue data to enable a robust evaluation of human and environmental risks [60].

Considering the biopesticidal EOs available on the market, their use is not without concern, and their safe application necessitates thorough toxicological evaluation. For instance, the EFSA assessed clove oil for use as a soil-applied nematicide on greenhouse tomatoes and cucumbers in addition to its post-harvest use as a fungicide and bactericide [55]. Clove oil was found to be effective against root-knot nematodes, and no critical issues were identified regarding its properties and formulation. However, uncertainties remain in mammalian toxicology and consumer exposure, as it is unclear whether eugenol toxicological reference values entirely apply to clove oil. Additionally, residue data in crops and drinking water are insufficient to complete the consumer risk assessment. Environmental fate data were generally sufficient, though the potential presence of methyleugenol in soil and groundwater could not be ruled out. Ecotoxicological risks to non-target organisms were considered low under the proposed greenhouse use. Overall, although clove oil exhibits pesticidal efficacy and low ecological risk in greenhouses, substantial uncertainties in toxicology and residues prevent a complete consumer risk assessment [55].

Orange oil (*Citrus aurantium* var. *dulcis* peel oil) is increasingly used as a botanical pesticide due to its primary active component, D-limonene, which exerts insecticidal and antifungal effects mainly through physical and contact mechanisms [118]. Its broad-spectrum activity and biodegradability make it an attractive option for organic agriculture. EFSA’s peer review of orange oil concluded that it exhibits low acute toxicity and does not require setting of reference values such as ADI or ARfD; thus, no MRLs were necessary given its characteristics and representative uses. The analytical methods for residues and detailed environmental fate studies were not mandated for regulatory risk assessment; however, data gaps in environmental toxicity and mixture complexity remained, underscoring unresolved uncertainties in the ecotoxicological profile. Generally, the regulatory assessment did not identify unacceptable risks for approved uses [56,119].

Tea tree oil (*Melaleuca alternifolia*) has been incorporated into biopesticide formulations in several non-EU markets, where it is valued for its insecticidal, fungicidal, and miticidal properties [120]. Its non-approval in the EU provides a useful case for comparing regulatory approaches and illustrates factors influencing the acceptance of botanical active substances. Tea tree oil has been proposed as a potential alternative to conventional pesticides due to its antifungal and insecticidal efficacy and its rapid environmental degradation [121]. However, EFSA’s peer review identified significant scientific uncertainties. In its 2012 assessment, EFSA highlighted concerns regarding the representativeness of the test material, the absence of clearly defined residues, and limitations in the available analytical methods [122]. These issues were not resolved in the subsequent confirmatory data evaluation, which concluded that critical data gaps remained, preventing the completion of consumer dietary risk assessment and exposure assessments for operators, workers, bystanders, and residents. In particular, insufficient information was available on the toxicological relevance and residue behavior of key impurities, including methyleugenol [123]. In parallel, EFSA FEEDAP Panel evaluations, conducted in a non-pesticide regulatory context, have identified tea tree oil as a skin and eye irritant and a dermal and respiratory sensitizer. It is also classified as a reprotoxic substance (category 1B) [124]. Taken together, these findings indicate that, despite its frequent perception as a promising botanical alternative, its safe use requires controlled formulation, appropriate handling measures, and further targeted research to address persisting toxicological and exposure uncertainties. Consequently, tea tree oil has not been approved as a PPP under Regulation (EC) No 1107/2009. More broadly, this case illustrates that while certain botanical substances may be approved under specific conditions, such approvals often depend on risk management measures and confirmatory data requirements to address remaining uncertainties. This regulatory context underscores both the potential value of biopesticides as safer alternatives and the ongoing need for robust, standardized data to ensure their safe use in modern agriculture.

A critical challenge in evaluating the potential effects of biopesticides on human health is the analytical complexity of detecting and quantifying their active ingredients [125]. Of particular concern is the possible presence of undeclared, insufficiently characterized, or unknown constituents in commercial formulations, which introduces additional uncertainty into both environmental and health risk assessments. For instance, a recent study evaluating commercial biopesticides derived from plant extracts and essential oils showed that these products contain a wide variety of volatile and non-volatile constituents, far beyond the declared active ingredients [126]. Products formulated with orange oil, cinnamon, or other EOs contained the highest number of detectable compounds, dominated by monoterpenes and sesquiterpenes. However, in addition to targeted compounds, dozens of other compounds were identified using untargeted approaches. In addition, several impurities, including dibutyl phthalate and butylated hydroxytoluene, were detected, suggesting contamination, formulation additives, or degradation products [126]. Although manufacturers are required to comply with regulatory frameworks [12], in practice, botanical biopesticides often contain complex mixtures of secondary metabolites, natural impurities, degradation products, and formulation additives that are not always disclosed or analytically characterized. This issue presents a considerable challenge for regulators, as it complicates hazard identification and makes it difficult to link specific components to biological activity or toxicity. From a public health perspective, the presence of uncharacterized constituents introduces significant uncertainty into exposure and risk assessments, since potentially bioactive or harmful compounds may remain undetected, unquantified, and therefore unassessed. Consequently, improving analytical characterization and ensuring stronger regulatory requirements for disclosure and quality control are essential for the reliable evaluation of health risks associated with botanical biopesticides.

Current analytical control of biopesticides increasingly relies on advanced high-resolution mass spectrometry workflows, including Ultra-high-performance liquid chromatography (UHPLC) and gas chromatography (GC) coupled to hybrid high-resolution mass spectrometry (HRMS), which enable simultaneous targeted and non-targeted screening of complex botanical formulations [126]. Comprehensive two-dimensional chromatography, GC×GC-MS (gas chromatography–mass spectrometry), has further improved the separation of volatile constituents and the detection of impurities in essential-oil-based products [127]. Rapid, minimal-preparation ambient ionization techniques such as desorption electrospray ionization (DESI-MS) and direct analysis in real time (DART-MS) facilitate fast screening and identification of adulterants or unknown components [128]. Additionally, Raman spectroscopy and Surface-enhanced Raman spectroscopy (SERS) have emerged as highly sensitive tools for detecting plant secondary metabolites, nanoparticle-based formulations, pesticide residues, and heavy metal residues [129]. The most common techniques used for the detection and quantification of botanical biopesticides in soil samples are GC-MS, HyDest (Hydrodistillation), LC-MS (liquid chromatography–mass spectrometry), SPME (solid-phase extraction), QuEChERS (Quick, Easy, Cheap, Effective, Rugged, and Safe), SLE (solid–liquid extraction), and UAE (ultrasound-assisted extraction) [52]. Together, these emerging technologies significantly improve detection capabilities but still face practical, technical, and interpretative constraints that limit their routine regulatory application. For instance, UHPLC-HRMS and GC-HRMS generate large, complex datasets that require sophisticated data-processing pipelines, expert interpretation, and extensive spectral libraries, which remain incomplete for many plant metabolites and formulation co-ingredients [130].

As noted earlier, in addition to organic farming, the use of botanical biopesticides alongside conventional pesticides is increasingly incorporated into IPM strategies to reduce reliance on synthetic pesticides [131]. However, as chemical pesticides continue to dominate global crop protection, a complete transition toward biopesticide-based strategies is therefore expected to be gradual, and the combined use of biological and chemical products is likely to persist and potentially increase [132]. Although the use of synthetic pesticides and the implementation of biopesticides have been widely documented, much less is known about their combined application, the interactions that may arise between them, and the potential agronomic or health implications of such mixed-use practices [133]. Evidence from field and laboratory studies indicates that certain combinations of botanical and chemical pesticides can produce synergistic or at least compatible effects [2]. For instance, the use of hybrid formulations, such as Regev, which contains tea tree extract and difenoconazole, illustrates how botanical–chemical mixtures can provide complementary modes of action and support pest resistance management [134,135]. Synergistic interactions are even more explored as a strategy to reduce overall insecticide inputs [136]. Numerous studies have shown that EOs and their constituents can potentiate the toxicity of conventional insecticides, including pyrethroids [137,138,139], organophosphates [138,139,140], neonicotinoids [141], and pyrrole insecticides, such as chlorfenapyr [136]. However, despite successful integration examples, many biopesticides still exhibit incompatibilities with certain synthetic pesticides, requiring sequential application schedules [2]. A commonly employed strategy involves applying a rapidly acting synthetic pesticide to achieve immediate pest reduction, followed by a biopesticide to provide longer-lasting control. This complementary approach can improve overall efficacy, slow the development of resistance, conserve beneficial organisms, and support ecological stability [2].

However, from a health-risk perspective, co-exposure to multiple pesticides may lead to additive or synergistic toxicological effects that differ from those observed for individual substances. The simultaneous presence of multiple pesticides may lead to interactions that result in partial detoxification (when antagonism or inhibition occurs) or in an increase in toxicity due to synergism or additive effects (“cocktail effect”), even when each compound is present at levels below toxicity reference values [142,143]. Interactions with residues of conventional pesticides or other agrochemicals in soil can alter degradation pathways, potentially leading to the formation of metabolites of unknown toxicity. Indeed, botanical biopesticides are often assumed to degrade rapidly, yet their persistence varies widely with soil properties such as pH, organic matter, clay content, temperature, and moisture [52,144,145,146,147]. Microbial metabolism is usually the dominant degradation pathway, but the persistence and bioactivity of many degradation products remain poorly characterized. This gap is critical, as degradation products may differ substantially from the parent compound in terms of persistence, efficacy, and effects on non-target organisms; some may even be more persistent or toxic than the parent compound [148]. These unknown or insufficiently characterized metabolites may also interact with other pesticides present in mixtures, thereby complicating health and environmental risk assessments. Moreover, volatile compounds emitted by plants can interact with pesticides after they are applied to crops or transported in surface water, forming a range of products that may be even more hazardous than their precursors [149,150]. A recent study investigated photochemical reactions between pesticides (chlorothalonil and imidacloprid) and thyme volatiles (thymol, α-pinene, 3-carene, and linalool), both in solution and directly on plant leaves under simulated solar irradiation [149]. As a result of these interactions, transformation products of potential toxicological concern were formed, which differ from those expected from pesticide photodegradation alone. These findings underscore the importance of expanding our understanding of complex processes that affect multiple environmental compartments.

The uncertainties surrounding pesticide mixtures become even more complex when pesticides are delivered through advanced technologies. Recent advances in pesticide formulation mainly rely on encapsulation technologies to improve target specificity and pesticide release [52,151]. Polysaccharides are frequently used as encapsulating materials due to their biodegradability, biocompatibility, and chemical versatility, which allows for functional modification to enhance stability and control pesticide release [152]. Nevertheless, polysaccharides may possess inherent antimicrobial properties and can interact synergistically or antagonistically with botanical active ingredients [52]. Furthermore, nanotechnology-based encapsulation is increasingly being explored to enhance the stability and controlled release of botanical biopesticides, addressing limitations such as rapid degradation, volatility, and inconsistent field performance [153,154,155,156]. Nanoformulations usually employ nanocarriers, such as lipids, chitosan, proteins, silica/clays, polymers, or biomolecules, which can significantly improve bioavailability and prolong the pesticidal activity of botanicals [157]. Nanoemulsions, which consist of nanoscale oil–water droplets, offer similar advantages by increasing the dispersion and potency of botanical biopesticides [158,159]. However, the development of these technologies introduces new levels of uncertainty and potential risks. Particle size, surface functionalization, and environmental aging considerably influence the transport and toxicity of nanoparticles (NPs) [160]. Smaller NPs are more mobile and reactive, potentially increasing plant uptake and the risk of inducing oxidative stress, impaired root development, and reduced germination [161]. Additionally, nanoparticle-soil microbiome dynamics remain insufficiently characterized, raising concerns about effects on non-target organisms, trophic transfer, and cumulative toxicity [160]. Environmental aging of NPs alters their physicochemical properties, often reducing their mobility and toxicity, but sometimes new reactive or persistent forms may occur [162]. Although laboratory studies on nano-formulated pesticides have shown encouraging results, substantial knowledge gaps persist regarding their environmental fate, chronic exposure, and effects on soil biota and non-target organisms [160].

Given the complexity of environmental and dietary exposure to multiple compounds, human biomonitoring (HBM) has become a crucial tool for assessing real-world pesticide exposure, enabling the evaluation of internal body burden from various sources and exposure routes, including environmental, occupational, and dietary [163]. Numerous studies worldwide use biomarkers, typically pesticide metabolites in urine, blood, serum, or other biological samples, to evaluate internal exposure in general populations and occupationally exposed groups [164,165]. The European HBM initiative, HBM4EU, is a joint effort of 28 countries, the European Environment Agency, and the European Commission, and co-funded under Horizon 2020. The HBM4EU survey, conducted between 2014 and 2021 across five European countries, has provided evidence of widespread exposure and common co-occurrence (mixture exposure) among European populations [166,167,168,169,170,171]. At least 46 pesticide residues or metabolites were detected in adults and children [172], with 84% of samples containing two or more pesticides [173]. Notably, all monitored pesticides were found at higher concentrations in children than in adults [171]. Conversely, consumption of organic fruit and vegetables has been linked with reduced pesticide body burdens in both children and adults [174]. Results also show that the general population is primarily exposed to pesticides through diet. For some individuals or specific groups, however, substantial exposure may also arise from occupational and residential settings, affecting both workers and the general public. Initiatives such as the European HBM4EU project aim to incorporate human biomonitoring data into regulatory processes to strengthen chemical risk assessment, addressing both the general population and occupationally exposed groups, while also addressing the challenges of applying HBM data to broader risk-management strategies [163].

The ongoing transition toward more sustainable agricultural practices, including the increased adoption of organic production systems, has contributed to a measurable decline in the internal exposure to certain conventional pesticides, as demonstrated across numerous human studies [175,176,177]. However, these studies usually do not assess exposure to botanical biopesticides [178], and to the best of our knowledge, direct human exposure data for botanical biopesticides are missing. Since HBM programs play a pivotal role in providing data to guide policy responses to environmental and public health risks, developing and advancing analytical methods for detecting and evaluating biopesticides is crucial and should be integrated into future HBM initiatives [164]. Additionally, recent studies have highlighted the need to assess adjuvants, counterions, and other co-formulants in exposed populations [179] and to consider mixture effects [180]. Advances in chromatography–mass spectrometry for urinary analysis have enabled the development of multi-residue methods that simultaneously quantify numerous pesticide compounds across different chemical classes. These advances are valuable for investigating health risks associated with combined exposures to pesticides and other contaminants, thus supporting a more integrated understanding of human biomonitoring [164,171]. In this context, expanding toxicological datasets for biopesticides and coordinated approaches that strengthen international collaboration and biomonitoring programs are essential. Such efforts enhance resource efficiency and substantially improve the effectiveness of initiatives to assess human health risks.

## 4. Routes of Human Exposure to Botanical Biopesticides and Associated Health Risk

As for the synthetic pesticides, human exposure to biopesticides can occur through several routes, including oral (dietary and non-dietary) intake, dermal contact, and inhalation, depending on the patterns of use and environmental behavior of the active substances [181]. The extent and duration of these exposure pathways are closely influenced by the environmental fate of biopesticides, particularly their dissipation rate and half-life in soil, water, and on plant surfaces [182]. Although many biopesticides exhibit rapid degradation, low persistence, and minimal residues on treated crops [70], these features do not preclude exposure, especially shortly after application, underscoring the importance of integrating exposure routes with dissipation dynamics in human health risk assessments.

The half-life of botanical biopesticides varies widely depending on the active substance, environmental conditions, and formulation. The reported period required for 50% dissipation (DT_50_) of botanical biopesticides often ranges from several hours to several days in soil, water, or on plant surfaces; however, complete dissipation data are available for only a limited number of botanical biopesticides and often show considerable variation across databases and the scientific literature. Differences may arise from varying experimental conditions, formulations, environmental settings, and data quality [182,183]. In general, compounds such as azadirachtin, pyrethrins, and essential oils degrade relatively quickly due to photolysis, microbial activity, and hydrolysis [49,70]. For example, azadirachtin is rapidly broken down by sunlight and microbial activity in soil, water, and on plant surfaces. The reported soil half-life is 3–44 days, while in water, it ranges from 48 min to 4 days. In foliage, azadirachtin is also short-lived, typically persisting for 1 to 2.5 days [184]. Pyrethrin 1, one of the main constituents of pyrethrins, degrades quickly when exposed to sunlight, with a half-life of 12 h in water and on soil surfaces. On plant leaves such as tomato and potato, it dissipates rapidly, with less than 3% remaining after five days. In the absence of light, pyrethrin 1 breaks down more slowly in water, with a reported half-life of 14–17 days, and its degradation is further reduced in acidic conditions. In aquatic environments, pyrethrins have low solubility and tend to bind strongly to sediments, with half-lives ranging from 10.5 to 86 days. In surface soils, pyrethrins are rapidly degraded by microorganisms, with half-lives typically ranging from 2.2 to 9.5 days [185]. The half-life of essential oils is often less than 1 day due to volatility and photodegradation [186]. For example, for clove oil, the air half-life is 0.01 h, the soil half-life is 21.5 h, and the water half-life is 78.4 h [187]; another database reported a soil DT_50_ of 0.99–2.09 days [99]. Citronellol, citronellal, and geraniol, the primary constituents of citronella oil, undergo rapid degradation in air, with reported half-lives ranging from approximately 38 min to 3.2 h. In aquatic systems, they evaporate from the water surface at a moderate rate [188]. For thymol, soil DT_50_ = 0.7–2.6 days [99]. For eugenol, soil DT_50_ = 0.5–2.4 days, aqueous photolysis DT_50_ = 9.2 days, and aqueous hydrolysis DT_50_ = 120 days [99]. Tea tree oil is also reported to degrade rapidly in the environment. Studies show that up to 90% of applied tea tree oil volatilizes within 24 h, and residue analyses report no detectable levels of several major constituents 48 h after application. However, its soil half-life is not established [121]. Likewise, DT_50_ values for orange oil and numerous other essential oils, as well as their ingredients, are either incomplete or unavailable.

The lack of reliable DT_50_ data creates uncertainty in assessing human exposure to biopesticides [182]. Without robust dissipation parameters, it is difficult to accurately predict residue persistence in food crops, potential environmental accumulation, or the timing of safe harvest intervals. Determination of pesticide half-life is a key parameter for establishing pre-harvest intervals (PHIs) and ensuring compliance with MRLs to protect consumer health [189,190]. For accurate PHI setting, it is important to note that pesticide dissipation is influenced not only by the chemical properties of the compound but also by environmental conditions, such as temperature, moisture, rainfall, sunlight, and biota, which vary across regions [191]. These constraints may lead to either underestimation or overestimation of exposure and associated human health risks. Consequently, limited DT_50_ information underscores the need for robust risk assessments and additional experimental data to support evidence-based regulatory decisions. Generally, biopesticides are considered safer than conventional pesticides, with low PHI and reduced restricted-entry intervals [192]. Table 3 presents a comparative overview of selected botanical and synthetic pesticides approved for use in the EU [50]. Parameters included formulation type, the application method and frequency, PHI, and established MRLs, with data obtained from the EU Pesticides Database [50]. This structured comparison allows evaluation of potential exposure pathways and regulatory considerations relevant to both consumer and occupational risk assessment. Across the uses examined, botanical biopesticides may require comparable or more frequent applications, resulting in higher cumulative seasonal application rates. Nevertheless, active substances such as thymol, eugenol, geraniol, clove oil, and orange oil are not subject to specific MRL requirements, reflecting their lower toxicological concern compared with conventional synthetic pesticides.

Dietary intake is the main route of exposure for the general population, due to residues on treated crops [193]. When used according to good agricultural practices (GAPs), dietary exposure to botanical biopesticides is generally considered low [194]. The EFSA reported that in 2022, residue quantification and MRL exceedance rates were generally lower in organic food than in conventionally produced food, mainly due to copper, which is authorized in organic production but is also used in feed and fertilizers. A total of 6717 organic food samples were analyzed in 2022, representing 6.1% of all samples. Most samples (79%) contained no quantifiable residues, while 18.6% had residues at or below the MRLs, 2.4% exceeded the MRLs, and 1.4% were classified as non-compliant. The most frequently quantified substances were copper compounds, bromide ion, and chlorates, with copper compounds accounting for the majority of MRL exceedances. No specific consumer health concerns related to botanical PPPs were reported. In 2023, the exceedance rates of MRLs in organic food were lower than in 2022 [195]. Occasional detection of unauthorized pesticides in organic farming may result from spray drift, environmental contamination, post-harvest handling, or mislabeling. Consequently, EFSA recommends that Member States clarify the sources of unauthorized residues detected in organic products and expand analytical screening, thereby improving the reliability of monitoring data and strengthening the integrity of organic food control systems [194]. Additionally, although isolated ARfD exceedances were reported for bio-derived active substances, such as abamectin and spinosad, in a small number of samples, EFSA concluded that overall dietary exposure does not pose a health concern for consumers. These findings reflect sporadic non-compliance rather than a general risk associated with bio-derived pesticides. In this context, adherence to recommended use patterns, including PHIs and GAP, is essential to keep dietary risks low. Consuming crops before the recommended PHIs can increase dietary exposure, potentially leading to acute or sub-acute toxic effects in consumers [196].

Chronic exposure to pesticides poses significant health risks, particularly for operators and workers in high-exposure settings such as agriculture, where contact with concentrated products may occur through dermal absorption and inhalation during preparation, mixing, and application [181]. Dermal exposure represents the dominant route for operators and re-entry workers, especially when using formulations based on EOs, which may exhibit high dermal absorption [197,198]. Inhalation exposure may also be relevant during the application of volatile botanical products or formulations generating aerosols, vapours, or fine particulates, including EOs, wettable powders, and emulsifiable concentrates. In addition, bystanders and residents may be exposed through spray drift, aerosol dispersion, or contact with treated surfaces. Accidental, non-dietary oral exposure, although less frequent, may occur through hand-to-mouth behavior, particularly in children [19].

From an occupational perspective, the potential human health risks associated with botanical biopesticides depend on the type of active substance, formulation, and application method [83]. A key advantage of EO-based biopesticides is their rapid volatilisation, which generally results in minimal residues in food and soil [199]. However, this often requires higher application rates or repeated treatments, potentially increasing environmental exposure [200]. Accordingly, encapsulation and other formulation strategies have been developed to enhance efficacy and thereby reduce unintended exposure. Solid or encapsulated formulations generally reduce direct contact and volatility by slowing the release of active compounds [52]. However, toxicological properties of encapsulating materials, additives, or wetting agents should also be considered in risk assessments [52,83]. Due to their volatility and lipophilicity, liquid EO-based formulations pose the highest exposure risk as their compounds may penetrate the skin and respiratory mucosa, potentially inducing harmful effects [83,201]. The use of PPE and adherence to safety guidelines are therefore critical to minimizing user risk [181].

Beyond direct occupational exposure, EO-based biopesticides may contribute to airborne drift due to the volatility of their constituents. Studies in indoor environments indicate that evaporating EOs can increase concentrations of volatile organic compounds (VOCs), carbon monoxide, and carbon dioxide, and may promote the formation of secondary pollutants, such as formaldehyde and secondary organic aerosols (SOA), through reactions with oxidants and ozone [202,203]. Direct studies on the atmospheric reactions of EO-based biopesticides are scarce. Still, extensive research on VOCs, particularly biogenic terpenes, shows that terpene-rich emissions undergo atmospheric oxidation, forming low-volatility products that contribute to SOA and other secondary pollutants [204,205]. Oxidation of monoterpenes such as α-pinene yields oxidation products that readily partition into the particle phase, constituting a substantial fraction of SOA mass in the atmosphere [206]. Sesquiterpene oxidation products have also been shown to contribute significantly to aerosol particle composition under ambient conditions, despite lower gas-phase concentrations [207]. Studies of indoor essential oil volatilisation further indicate that evaporated EO constituents can emit measurable particulate matter (PM), suggesting that botanical VOCs may contribute to aerosol formation [208]. Pesticides released into the environment, including their active substances and formulation components such as solvents and adjuvants, may undergo chemical and biological degradation, generating products that may be more hazardous than the parent compounds [209]. These degradation products can further interact with natural VOCs emitted by treated crops, leading to the formation of secondary air pollutants, including ozone and fine PM. Ozone, one of the most important gaseous pollutants and greenhouse contributors, poses well-documented risks to human health, particularly for vulnerable populations, and exerts phytotoxic effects on some crops [210,211,212]. Recent experimental studies conducted in the large outdoor European PHOto-REactor (EUPHORE) demonstrated that synthetic pesticides, chloropicrin and formulated chlorpyrifos, when reacting with terpenes representative of orange tree emissions, promoted significant SOA formation during photolysis and ozonolysis [150]. In addition, their interaction with VOCs enhanced ozone formation, which, in turn, increased PM levels through further terpene oxidation, indicating potential atmospheric impacts at both local and global scales [150]. Such aerosols can induce short-term acute effects like bronchitis and long-term chronic respiratory irritation and inflammation, which can lead to cancer [213]. Moreover, they can be used by viruses and bacteria as transmission vectors, thereby generating serious public health problems [214]. Notably, even formulation additives regarded as “inert” can contribute to secondary pollutant formation following atmospheric degradation [150]. These findings highlight the need for improved understanding of such interactions and for their integration into environmental fate and exposure models, as the combined presence of pesticide formulations and terpene emissions in the atmosphere can substantially contribute to the formation of secondary pollutants [150].

**Figure 2:**
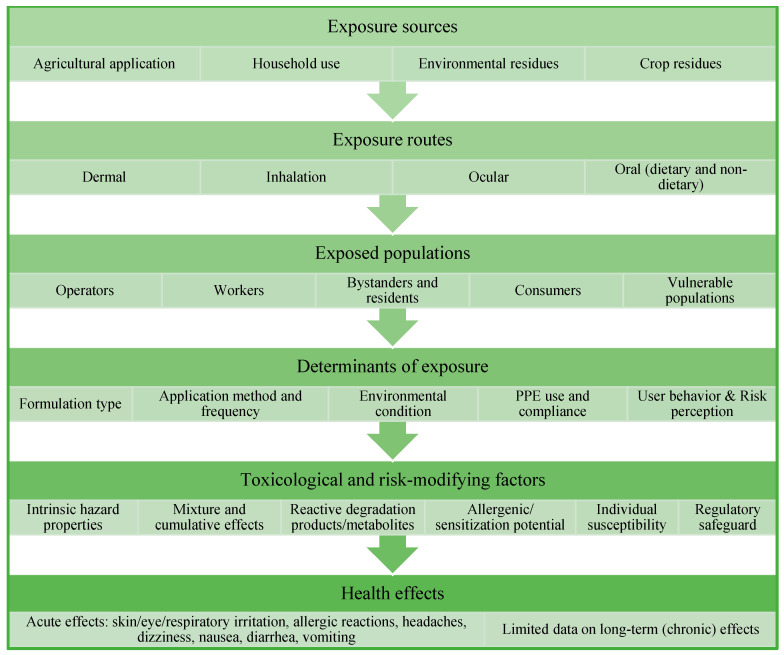
Schematic overview of human exposure sources, exposure routes, at-risk populations, and key determinants influencing exposure levels and health outcomes associated with botanical biopesticides.

Even low-toxicity or rapidly degrading substances, such as botanical biopesticides, can contribute to cumulative exposure risks when used alongside other pesticides or under frequent/long-term exposure scenarios. As discussed, botanical biopesticides can interact with other pesticides and agrochemicals, potentially forming reactive intermediates. Such interactions can occur in soil, water, or on plant surfaces, where multiple compounds coexist, and may generate products that are not assessed in standard risk assessments. The likelihood and significance of such interactions remain largely unexplored. However, their potential to cause unforeseen toxicological or environmental effects highlights the need for further research on combined exposure to multiple pesticides and other agrochemicals. A comprehensive schematic overview of the major exposure sources, exposure routes, the populations susceptible to exposure, and the key factors influencing exposure levels and potential health outcomes associated with botanical biopesticides is presented in Figure 2.

## 5. Public Risk Perception of (Bio)pesticides

The scientific literature addressing risk perception related to pesticide use has primarily focused on synthetic pesticides, emphasizing their well-documented adverse effects on human health and the environment. In contrast, studies examining the risk perception associated with biopesticides, particularly their potential harmful effects when used outside recommended conditions, remain limited. Farmers and consumers commonly perceive biopesticides as inherently safe or low-risk alternatives to synthetic pesticides [215]. However, this perception may overlook the fact that biopesticides, including botanical formulations, may pose risks to human health and the environment if not applied in compliance with safety recommendations. This disparity between perceived and actual risk highlights the need for improved risk communication and training, emphasizing that biopesticides, like conventional pesticides, require accurate risk assessment, regulatory governance, and adherence to GAP to ensure human and environmental safety. EFSA monitoring data indicate that occasional residue findings and MRL exceedances in organic food products in the EU are often linked to cross-contamination, spray drift, or deviations from application rates and preharvest intervals [194]. These observations indicate that current use patterns continue to result in measurable contamination and exposure risk despite regulatory efforts. These findings highlight that compliance with good agricultural practice remains essential to ensure that the use of authorized PPPs does not compromise human health.

The adoption of biopesticides has not yet become dominant in agricultural practice [132], making effective risk communication a key policy consideration, necessary to support their safe and sustainable integration into agricultural systems. Evidence from numerous studies indicates that farmers frequently fail to fully adhere to recommended application rates, pre-harvest intervals, and safety measures for synthetic pesticides [131]. These practices raise concerns about how recommendations for biopesticides will be implemented. If compliance with well-recognized high-risk substances remains suboptimal, there is a risk that guidance for biopesticides will be followed even less rigorously.

In this context, evidence from a recent Chinese survey provides an illustrative example of how risk perception translates into actual application behavior and adherence to recommended practices [132]. Based on self-reported use patterns, farmers in rice–crayfish integrated systems were categorized as chemical pesticide users (72.13%), biopesticide users (14.27%), or mixed-use farmers who applied both product types (10.5%) [132]. Across all user groups, insecticidal efficacy was the primary determinant of pesticide choice, reflecting strong concerns about yield loss. In contrast, toxicity considerations played a secondary role in pesticide selection, with mixed-use farmers expressing the lowest level of concern. Attributes such as insecticidal spectrum and effectiveness duration also influenced decision-making. Notably, biopesticide users placed greater emphasis on these characteristics than chemical-only or mixed-use farmers, likely reflecting labor constraints and economic considerations. Narrow insecticidal spectra and shorter effectiveness periods may increase production uncertainty and labor demands, particularly in contexts of limited household labor and high supervision costs for hired workers [216]. Approximately one-quarter of chemical pesticide users reported no specific selection criteria, indicating potentially indiscriminate use. Regarding application practices, 66% of chemical pesticide users based their application rates on personal experience or advice from pesticide vendors. Nearly half of mixed-use farmers adopted neighboring farmers’ practices, underscoring the influence of informal knowledge networks. Among biopesticide users, 18.9% reported following guidance from agricultural technicians; however, adherence to label instructions was low across all groups, consistent with evidence that farmers rarely consult pesticide labels before application [217]. Attention to application timing was generally low, particularly among chemical pesticide users who frequently disregarded crop growth stages, potentially increasing exposure risks and reducing pest control efficiency [132].

A study in Greece examining farmers’ behavior regarding pesticide use, especially their willingness to adopt lower-risk pesticides, found that most respondents (76%) preferred using all available pesticides rather than lower-risk options. Only 24% expressed a preference for lower-risk products. Older farmers and those managing larger farms were less likely to adopt lower-risk pesticides. In contrast, younger and more educated farmers, as well as those applying pesticides more frequently, showed a greater willingness to use lower-risk alternatives. The likelihood of choosing lower-risk options increased significantly among trained farmers and those actively seeking pesticide information. The findings also reveal that many farmers lack sufficient knowledge about proper handling and toxicity. Most farmers were not interested in being informed about pesticides, and almost half of them perceived them as non-harmful substances. Even among those with relatively high literacy, label instructions are often ignored [218]. Similarly, a study of Irish farmers investigating compliance with legally required pesticide-use practices highlighted that patterns of pesticide application strongly influence risks to both users and the environment. Using an anonymous online questionnaire, 76 farmers across diverse agricultural sectors reported on their adherence to safety and regulatory guidance. While overall compliance was relatively high, significant unsafe practices were identified. Nearly half of the respondents admitted to not consistently using PPE. Moderate non-compliance with bee-protective measures was observed, and some farmers reported not emptying or cleaning spray tanks between applications, actions that can harm pollinators, soil organisms, and water quality. A small number acknowledged practices that pose a potential for serious watercourse contamination [219]. Collectively, these findings highlight the need for improved training, extension support, and risk communication to strengthen farmers’ decision-making and promote safer and more effective pesticide use.

Unsafe pesticide use is reported more frequently in low- and middle-income countries, reflecting structural, regulatory, and resource constraints. A substantial proportion of farmers receive little or no formal training and often fail to use PPE or follow safety instructions [220]. Limited awareness of pesticide toxicity is commonly associated with misuse, including excessive application rates and inappropriate timing of treatments [221]. For example, surveys in Bangladesh indicate widespread unsafe practices, with the majority of farmers failing to take proper precautions during pesticide application, storage, and transport [222]. In many cases, pesticides are applied at doses far above recommended levels, and pesticide residues are frequently detected in vegetables, with a substantial proportion exceeding MRLs. Pesticide exposure contributes to a high burden of poisoning cases annually, affecting both agricultural workers and the general population through contaminated food and water [222]. In many low-income countries, enforcement of pesticide regulations and farmer guidance through extension services remains limited, often relying on non-governmental organizations [223]. Local produce is rarely monitored for residues, unlike export crops, which can influence farmers’ and consumers’ perceptions of safety. Studies in Uganda reveal that nearly a quarter of tomato growers were unaware of the health risks of spraying near harvest, while about half applied pesticides within a week of harvest or even at harvest [224,225]. These practices were primarily motivated by perceived benefits, such as extending shelf life or enhancing product appearance, illustrating how risk perception, knowledge gaps, and economic incentives drive behaviors that may increase consumer exposure to pesticide residues. This highlights the need for interventions that address both awareness and practical decision-making in agricultural practices [223]. Studies have shown that years of formal education, quality-oriented production goals, commercial production orientation, knowledge and experience in pesticide application, and government supervision are positively and significantly associated with compliance with pesticide guidelines and regulations. In contrast, farmers primarily driven by quantity-oriented production objectives are significantly less likely to comply with recommended pesticide use practices [226].

Perceptions of pesticides as a risk to human health largely depend on regulatory frameworks, education level, agricultural practices, access to information, and socio-economic factors [220,227]. These factors are interrelated, indicating that education can enhance knowledge, but economic limitations or a lack of accurate information may outweigh this effect, leading to unsafe practices despite awareness of risk [228,229]. In developed countries, consumers tend to have higher awareness of pesticide risks, stronger trust in regulatory systems, and more access to information on food safety. This often leads to greater concern about health and environmental impacts, stronger demand for residue monitoring, and a preference for alternatives such as organic products [230]. The survey of Greek consumers reveals a persistent concern about pesticide residues in food, alongside recognition of the role of pesticides in ensuring food security and economic stability. Attitudes are influenced by a mix of demographic and informational factors; still, the findings highlight a significant gap in effective risk communication between regulatory bodies and the general public [230,231].

**Figure 3:**
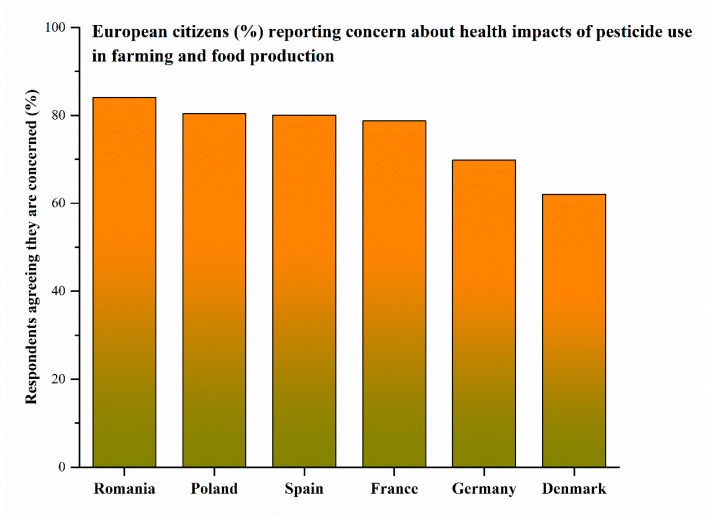
Concern about the health impacts of pesticide use in farming and food production among European citizens. Percentage of respondents who agreed with the statement: “I worry about how the use of pesticides in farming and food production is affecting my health or my family’s health,” based on a PAN Europe “Pesticides: Play It Safe!” survey. Only responses indicating agreement are presented [232].

The PAN Europe “Pesticides: Play It Safe!” survey, conducted in six EU Member States (Denmark, France, Germany, Poland, Romania, and Spain) in August 2023, reveals strong public concern about the impacts of pesticide use on human health, food safety, and the environment [232]. A substantial majority of respondents expressed concern about the healthiness of food (75.0%) and the environmental effects of farming and food production (79.5%) across the surveyed countries. A large majority of respondents expressed concern about the environmental effects of pesticides (81.8%) and agreed that pesticide use harms the environment (77.7%). Concern about the health impacts of pesticides was similarly elevated, with approximately 75.9% of participants expressing worry for themselves and their families. The strongest conviction is held in Romania (Figure 3). Trust in national governments to prioritize health and environmental protection in pesticide policy varied substantially across countries (relatively higher trust in Spain and Denmark, and notably lower trust in Romania, France, and Poland). The majority of respondents supported precautionary approaches, including tying EU financial support to lower-risk pest management and broader adoption of IPM. Many respondents also favor mandatory IPM and stronger regulatory safeguards. These findings align with broader EU public opinion trends reported in Eurobarometer surveys, which consistently identify pesticide residues as one of the top food safety concerns among European consumers [233]. In the Eurobarometer 2025 on food safety, pesticide residues were reported as the most frequently cited food risk among EU citizens (39%), despite a general trend of increasing confidence in EU food safety regulation. The persistent prominence of pesticides in consumer concerns highlights a broader pattern of risk perception that emphasizes both health and environmental dimensions, even as trust in regulatory institutions remains relatively high in the larger EU perspective [233].

In many developing countries, risk perception is often lower or more heterogeneous due to limited access to risk information, weaker regulatory oversight, and economic pressures that prioritize crop yield and income over perceived health risks [234].

Although many farmers underestimate the potential environmental and health risks, those with greater risk awareness are likely to adopt safer practices [221]. Consumers are usually aware of pesticide-related risks and are increasingly demanding organically produced food; however, consumer risk perception does not necessarily translate into consistent compliance at the production level. A study in Uganda illustrates a broader pattern in pesticide risk perception: awareness of potential health risks does not necessarily lead to reduced exposure [223]. Although consumers had a negative attitude towards pesticide residues, purchasing behavior was largely formed by limited access to safer alternatives rather than by risk knowledge alone. This gap between perception and behavior is well-documented in food safety research and highlights how structural factors, such as availability and affordability, often outweigh individual risk awareness.

The way farmers, regulators, and consumers perceive the risks of biopesticides directly shapes real-world exposure and safety outcomes, highlighting the necessity for targeted education, transparent regulation, and consistent communication to ensure safe use. The growing market uptake of biopesticides, driven by organic farming policies and consumer expectations, therefore necessitates proportionate investment in standardized toxicological datasets, harmonized analytical methodologies, and realistic exposure scenarios that reflect actual agricultural practices. In parallel, risk communication strategies should explicitly address the conditions under which biopesticides may pose human health or environmental risks, including misuse, repeated applications, co-exposure with other pesticides and contaminants, and mixture effects.

## 6. Conclusions

Botanical biopesticides pose distinct regulatory and toxicological challenges. They are widely promoted as safer alternatives to synthetic pesticides, yet their intrinsic chemical complexity and compositional variability introduce uncertainties that challenge conventional toxicological assessment frameworks. Unlike single-compound synthetic pesticides, botanical products are multi-component mixtures whose composition varies according to plant chemotype, extraction method, formulation, and manufacturing conditions. This variability complicates hazard identification, limits the reproducibility of toxicological findings, and challenges consistency in regulatory evaluation. Significant data gaps remain regarding chronic toxicity, mixture interactions, and real-world exposure scenarios. Additive or synergistic effects at low exposure levels are insufficiently characterized, and current testing strategies, largely developed for single active substances, may not fully capture mixture-related risks. Limited human biomonitoring data and constrained exposure assessment further increase uncertainty in evaluating long-term public health implications.

An important conclusion of this review is that perceived low risk can itself become a risk-modifying factor. The assumption that “natural” implies “safe” can reduce adherence to protective measures and influence user behavior, thereby indirectly increasing exposure. This sociobehavioral dimension of risk remains largely unaddressed in regulatory evaluation. We argue that the binary classification of pesticides as “synthetic” or “natural” obscures the spectrum of hazard and exposure potential. Botanical biopesticides should not be assumed to be inherently low-risk solely due to their natural origin, nor should they be evaluated simply as benign alternatives to synthetic active substances. Instead, they should be regulated as complex chemical mixtures that require robust compositional characterization, standardized toxicological assessment, and realistic exposure evaluation, comparable in scientific rigor to that applied to synthetic pesticides. Current regulatory frameworks address some of these issues; however, substantial gaps remain in toxicological standardization, exposure assessment, and post-authorization surveillance. Addressing these limitations will require coordinated interdisciplinary efforts, including improved analytical fingerprinting, harmonized toxicity testing strategies, expanded human biomonitoring, and integration of real-world exposure data.

Equally important are targeted risk communication and user training strategies that align stakeholder perceptions with scientific evidence. Evidence from both developing and developed regions indicates that behavioral factors, such as risk perception, training, compliance with protective measures, and access to reliable information, remain critical determinants of real-world exposure. Regulatory frameworks provide essential safeguards, yet actual risk reduction ultimately depends on proper implementation, monitoring, and user awareness.

Botanical biopesticides represent a valuable component of sustainable crop protection strategies. However, their safe and responsible use depends on evidence-based regulation, robust exposure data, and a realistic, informed understanding of their benefits and limitations.

## Figures and Tables

**Table 2:** Impact on human health and toxicological reference values for selected botanical biopesticides.

Botanical Biopesticide	ADI (mg/kg bw/Day)	ARfD (mg/kg bw)	AOEL (mg/kg bw/Day)	Impact on Human Health	Data Completeness	Reference
Pyrethrins (*Tanacetum cinerariifolium*)	0.04	0.2	NA	Pyrethrins are harmful if swallowed (H302), inhaled (H332), or absorbed through skin contact (H312). They may cause dermatitis and gastrointestinal issues. Potential thyroid and liver toxicants. No genotoxic concerns or evidence of carcinogenic potential were identified based on available data.	Data on local respiratory toxicity, dermal absorption, and irritation for all pyrethrin components are limited. Toxicokinetic and metabolic profiles of minor components are incomplete.	[50,54,99,100]
Azadirachtin (Margosa extract) (*Azadirachta indica*)	0.1	0.75	0.1	Azadirachtin extracts show low acute toxicity via oral, dermal, and inhalation routes. They are not skin or eye irritants, but may cause skin sensitization. Possible liver and thyroid toxicant. No evidence of reproductive, developmental, or neurotoxic effects.	Limited data are available on the toxicological characterization of individual components of azadirachtin extracts. Uncertainty remains regarding the representativeness and composition of batches used in toxicological studies, as well as the relative toxicity of the extract’s components. Limited data on the long-term toxicity or carcinogenicity.	[50,53,99]
Thymol (*Thymus vulgaris*)	0.03	0.08	0.4	Thymol shows moderate acute oral toxicity (H302) and is classified as corrosive (Skin Corr. 1B, H314). Skin, eye, and respiratory tract irritant. It may act as a skin sensitizer, cause gastrointestinal problems, central hyperactivity, and occasionally convulsions and coma. Possible renal, kidney, and liver toxicant. It may cause CNS or muscular contractions. No in vivo genotoxicity was observed.	Limited data regarding the relevance of impurities in test batches, including methyleugenol. Limited data on short-term, long-term, reproduction, or developmental toxicity.	[50,61,99,101]
Eugenol (*Syzygium aromaticum*)	1	/	1	Eugenol is harmful if swallowed (H302), irritant to skin and eyes (H315, H319), and a skin sensitizer (H317). Possible liver and kidney toxicant. It is genotoxic in vivo at very high doses. No carcinogenic or neurotoxic potential was observed.	Limited data for acute inhalation toxicity, assessment of the relevance of impurities including methyleugenol, and the representativeness of the tested technical material. Limited data on long-term reproductive toxicity.	[50,60,99]
Clove oil (*Syzygium aromaticum*)	1	/	1	Clove oil mainly contains eugenol (~80%), and it is harmful if swallowed. It can irritate the skin and eyes and act as a skin sensitizer. It may also be toxic to the liver. At high doses, it might cause acute respiratory distress and CNS depression.	Data gaps exist in the toxicological characterization of minor components, including methyleugenol, and in the applicability of eugenol reference values to clove oil. There is limited data on long-term reproductive or developmental toxicity.	[50,55,99,102]
Orange oil (*Citrus aurantium var.* *Dulcis*)	NA	NA	NA	Orange oil consists mainly of D-limonene (~94%), classified as a skin irritant and skin sensitizer.	Limited data on the relevance of all components in the technical material, operator exposure for greenhouse uses, metabolic fate after crop application, and insufficient background exposure information to complete operator, worker, and bystander risk assessments.	[50,56,99]

bw—body weight; NA—Not applicable (value not established due to insufficient data); /—ARfD not required (numerical value not set by regulatory authorities).

**Table 3:** Comparative performance and regulatory context of selected botanical and synthetic pesticides used against comparable target pests, under representative EU agricultural uses.

Pesticide/Formulation Type	Representative Crop	Target Pest	Application			Application Rate per Treatment kg a.s./ha (Max)	PHI (Days)	MRL (mg/kg)	Note	References
			Method	Number (Max) per Season	Interval Between Appl.					
Pyrethrins (botanical)/EC	Lettuce	Aphids *Nasonovia* *ribisnigri*	Spray	4	7 days	0.030	2	1	- non-systemic insecticide with repeated applications and short PHI, rapid responses to pest outbreaks. Established residue limits.	[50]
Flupyradifurone (synthetic)/SL			Spray	1 appl per 24 months	NA	0.125	10	6	- systemic synthetic insecticide with prolonged residue relevance, longer-lasting control, and strict application restriction. Established residue limits.	
Azadirachtin (Margosa extract) (botanical)/EC	Potato	Colorado Beetle *Leptinotarsa decemlineata*	Spray	1	NA	0.025	4	1	- botanical insecticide with low seasonal load. Established residue limits	[50]
Acetamiprid (synthetic)/SG			Spray	3	7 days	0.05	7	0.01	- systemic synthetic neonicotinoid with higher seasonal load, and more frequent applications. Established residue limits.	
* Thymol/Eugenol/Geraniol (botanical)/CS	Tab le and Wine grape	*Botrytis* *cinerea*	Spray	1–4	7 days	0.05–0.13 (E), 0.10–0.26 (G), 0.10–0.26 (T)	7	/	- multi-component botanical fungicide with repeated applications. No MRL requirement.	[50]
Fenhexamid (synthetic)/WG			Spray	2	7–14 days	0.75	14	15	- synthetic fungicide with less frequent applications. Established residue limits	
Clove oil (botanical)/EC	Pears	*Gloeosporium* spp. *Penicillium* sp.	Postharvestdrench	1	-	90–450 g/hL	-	/	- botanical fungicide for postharvest treatment. No MRL requirement.	[50]
Thiabendazole (synthetic)/SC			Postharvest drench	1	-	0.048	-	4	- systemic fungicide with established residue limits	
Orange oil (botanical)/ME	Tomato	Whitefly *Bemisia tabaci*	Spray	3	7 days	0.024 to 0.120	-	/	- non-systemic botanical with repeated applications, no defined PHI, and no MRL requirement.	[50,56]
Cyantraniliprole (synthetic)/SC			Hydroponic	1–4	7–14 days	0.1	1	1	- systemic diamide insecticide with repeated applications. Established residue limits.	

Commercial product names are not listed; the corresponding details are publicly available in the EU Pesticides Database [50]. * product contains eugenol (E), geraniol (G), and thymol (T). SG—water-soluble granule; ME—micro-emulsion; SL—soluble liquid; SC—suspension concentrate; WG—water-dispersible granules; CS—capsule suspension; NA—not applicable; /—MRL not set by regulatory authorities.

## Data Availability

No new data were created or analyzed in this study. Data sharing is not applicable to this article.
